# Host Immune Response to Clinical Hypervirulent *Klebsiella pneumoniae* Pulmonary Infections via Transcriptome Analysis

**DOI:** 10.1155/2022/5336931

**Published:** 2022-09-20

**Authors:** Langhuan Lei, Xiaoli Zhang, Rui Yang, Haiming Jing, Yue Yuan, Zhifu Chen, Qiang Gou, Zhuo Zhao, Jinyong Zhang, Xingyong Wang

**Affiliations:** ^1^Department of Critical Care Medicine, Children's Hospital of Chongqing Medical University, Chongqing 400014, China; ^2^Department of Clinical Hematology, College of Pharmacy, Third Military Medical University, Chongqing 400038, China; ^3^Department of General Surgery, Central Theater Command General Hospital of PLA, Wuhan 430000, China; ^4^National Engineering Research Center of Immunological Products & Department of Microbiology and Biochemical Pharmacy, College of Pharmacy, Third Military Medical University, Chongqing 400038, China

## Abstract

*Klebsiella pneumoniae* (*K. pneumoniae*), especially those with hypervirulence, is becoming a global concern and posing great threat to human health. Studies on individual immune cells or cytokines have partially revealed the function of the host immune defense against *K. pneumoniae* pulmonary infection. However, systematic immune response against *K. pneumoniae* has not been fully elucidated. Herein, we report a transcriptome analysis of the lungs from a mouse pneumonia model infected with a newly isolated *K. pneumoniae* clinical strain YBQ. Total RNA was isolated from the lungs of mice 48 hours post infection to assess transcriptional alteration of genes. Transcriptome data were analyzed with KEGG, GO, and ICEPOP. Results indicated that upregulated transcription level of numerous cytokines and chemokines was coordinated with remarkably activated ribosome and several critical immune signaling pathways, including IL-17 and TNF signaling pathways. Notably, transcription of cysteine cathepsin inhibitor (*stfa1*, *stfa2*, and *stfa3*) and potential cysteine-type endopeptidase inhibitor (*cstdc4, cstdc5*, and *cstdc6*) were upregulated. Results of ICEPOP showed neutrophils functions as the most essential cell type against *K. pneumoniae* infection. Critical gene alterations were further validated by rt-PCR. Our findings provided a global transcriptional perspective on the mechanisms of host defense against *K. pneumoniae* infection and revealed some unique responding genes.

## 1. Introduction

*Klebsiella pneumoniae* (*K. pneumoniae*) is a critical opportunistic bacterial pathogen and a frequent cause of life-threatening nosocomial or community infections, including bacteremia, pneumonia, liver abscess, and urinary tract infection [1]. Due to the steadily increase in antibiotic resistance and hypervirulent *K. pneumoniae* (hvKp) strains, *this bacterium* was listed as a top threat to public health by World Health Organization in 2017 [2], with reports of mortality rates up to 50% or higher [3]. Given the fact that a variety of virulence determinates were identified in this bacterium [4], studies focused on the host response to hvKp are relatively limited.

Several cytokines have been reported to be involved in mediating host defense against *K. pneumoniae infection.* Deficiency or impairment of TNFR1 [5], CCL3 [6], CXCL15 [7], leukotrienes synthesis [8], or nitric oxide production [9] in mouse impaired clearance of *K. pneumoniae*. In addition, intrapulmonary over-expression of CCL3 [10] and intratracheal instillation of CpG [11] promoted recruitment of neutrophils, *αβ* T cells, *γδ* T cells, and activated NK cells to the site of infection. Further, downstream of these stimulators, such as IL-23 [12], IL-17 [13], IL-12 [12], and IFN-*γ* [14], was also required for efficient eradication of *K. pneumoniae*. Besides, the role of type I IFN in host defense against *K. pneumoniae* infections was also reported recently [15]. Moreover, the role of pathogen recognition receptors and their downstream signaling pathways in host defense against *K. pneumoniae* infections were elucidated in mice deficiency of MyD88 [16], which is an indispensable hub for TLRs signaling except for TLR3 [17], and TRIF [16], a sole adaptor for TLR3.

Meanwhile, extensive immune cells were reported to participate in combating *K. pneumoniae* infection. In a murine model, monocytes, inflammatory macrophages, and dendritic cells were accumulated in lungs, but the numbers of alveolar macrophages were not altered by infection [18]. CCR2-deficiency mouse infected with *K. pneumoniae* showed reduction of all subsets of lung mononuclear phagocytes but not neutrophils [18]. Furthermore, eradication of the rodent-adapted and 4 clinical *K. pneumoniae* strains required either neutrophils or CCR2+ monocytes. Strikingly, neutrophil depletion did not impact clearance of a carbapenem-resistant strain, ST258. In contrast, depletion of CCR2+ monocytes significantly increased the mortality of mice infected with ST258 [19]. Detailed investigation revealed that the CCR2+ monocytes-mediated bacterial uptake and killing are enhanced by IL-17A, which is produced by innate lymphocytes stimulated with TNF [20].

The hvKp typically exists as hypermucoviscous phenotype characterized by increased expression of extracellular polysaccharides, which is a major virulence factor of *K. pneumoniae* [21]. Recently, we have isolated a clinical strain of *K. pneumoniae,* named YBQ, from the sputum of an acute pneumonia patient with *K. pneumoniae* infection. String test showed a greater than 5 mm “string” between an inoculating loop and a plated bacterial colony, which indicated a hypermucoviscous phenotype [22]. Challenge studies confirmed that YBQ is an hvKp since all mice died within 96 hours after infected with 5 × 10^6^ CFUs of YBQ; and the death was accompanied with high bacteria burden, cytokine storm and tissue damage in the lungs of infected mice [23]. Although a number of studies have revealed the function and mechanism of some host factors in *K. pneumoniae* infection, a global perspective on the host immune response in response to hvKp is needed. Herein, we proposed a transcriptome analysis of pulmonary infection with *K. pneumoniae* YBQ strain in mice, and the transcriptome data was further processed with KEGG, GO, and ICEPOP bioinformatic methods. These data may shed light on the immune response to K. pneumoniae infection *in vivo*.

## 2. Materials and Methods

### 2.1. Mice and Bacteria Strain

6 to 8-week-old female BALB/c mice were purchased from HUNAN SJA LABORATORY ANIMAL CO., LTD., raised under specific pathogen free conditions. All animal experiments were approved and carried out according to the guidelines of the Animal Ethical and Experimental Committee of the Third Military Medical University (Chongqing, Permit No. 2011–04). *K. pneumoniae* strain YBQ was isolated from the sputum of a patient with severe pneumonia [24].

### 2.2. Mouse Pneumonia Model

BALB/c mice were infected with *K. pneumoniae* strain YBQ as described previously [23]. In brief, YBQ was grown in LB medium to exponential phase (OD_600nm_ = 0.5 − 1.0), and then adjusted to final concentration. After anesthetized with pentobarbital sodium (1-1.25 mg/20 g), mice (n =10) were intratracheally challenged with different doses of strain YBQ (1.0 × 10^7^ CFUs, 5.0 × 10^6^ CFUs and 1.0 × 10^6^ CFUs, respectively) in a total volume of 20 *μ*l, and the control group was injected with an equal amount of PBS, the survival of mice was recorded daily for 14 days to determine the virulence of the strain. To determine bacterial burden, mice in each group (*n* = 3) were challenged with a sublethal dose (1 × 10^6^ CFUs/mice) of strain YBQ, lung tissue and blood were collected at 24 h, 48 h and 72 h after infection, respectively. Lung homogenates prepared in PBS and blood were plated at 10-fold serial dilutions on LB agar, and the colonies were quantified after 24 h of incubation at 37°C.

### 2.3. Histopathology Analysis

Forty-eight hours after infection, the lungs of the mice were harvested and fixed in 4% paraformaldehyde. Next, the lungs were dissected into 4 mm thick sections, embedded in paraffin, and stained with hematoxylin and eosin for microscopic examination.

### 2.4. RNA Extraction, Library Construction, and Sequencing

Lung total RNA was extracted using the TRIZol (Invitrogen) RNA extraction reagent. According to the instruction of VAHTS mRNA-seq V3 Library Prep Kit for Illumina, mRNA was purified and reverse transcribed into cDNA, and cDNA was further purified and enriched. The quantity and quality of cDNA libraries were determined by Agilent 2100 Bioanalyzer (Agilent), Agilent High Sensitivity DNA Kit (Agilent), Quantifluor-ST fluorometer (Promega), and Quant-iT PicoGreen dsDNA Assay Kit (Invitrogen), respectively. Illumina Novaseq 600 platform (Illumina, USA) was used for sequencing.

### 2.5. RNA-Seq Analysis

Cutadapt (v1.16) was used to filter the sequencing data to get high quality sequence for further analysis. Reference genome index was built by Bowtie2 (2.2.6), and the filtered reads were aligned to the reference genome using Tophat2 (2.0.14), the default mismatch was no more than 2. HTSeq (0.9.1) was used to compare the read count values on each gene as the original expression of the gene and then used FPKM to standardize the expression. DESeq (1.30.0) was used to analyze the genes of difference expression with screened conditions as follows: expression difference multiple ∣log_2_FoldChange | >1, significant *P*value < 0.05.

### 2.6. Immune Cell Typing

ICEPOP (Immune CEll POPulation) interactive web site: (https://vdynamics.shinyapps.io/icepop/) and Pythonpackage: (https://github.com/ewijaya/icepop) were used to perform *in-silico* analysis of immune cell population from differential gene expression data. The gene expression of immune cell types was obtained from two public datasets: ImmGen (http://www.immgen.org/) and IRIS (http://share.gene.com/share/clark.iris.2004/iris/iris.html). Different immune cell subtypes were grouped into 10 major cell types.

### 2.7. Quantitative Real-Time PCR

Lungs were homogenized in liquid nitrogen via Dounce Tissue Grinder. And total RNAs were extracted using TRIzol (Invitrogen). 1 *μ*g of total RNA was reverse transcribed into cDNA using PrimeScript RT reagent Kit (Takara). Quantitative real-time PCR was performed with SYBR Green on CFX96 (Bio-Rad). Relative gene expression levels were normalized to GAPDH as control and further to levels of mock-infected control samples (2^−ΔΔCT^).

### 2.8. Statistical Analysis

GraphPad Prism software (version 8.01) was used to analyze statistical data. Unpaired student's *t*-test was performed to compare two data sets. Data were presented as means ± SD.

## 3. Results

### 3.1. Acute Pneumonia Induced by *K. Pneumoniae* YBQ Reached a Turning Point at 48 Hours Post Infection

**Figure 1:**
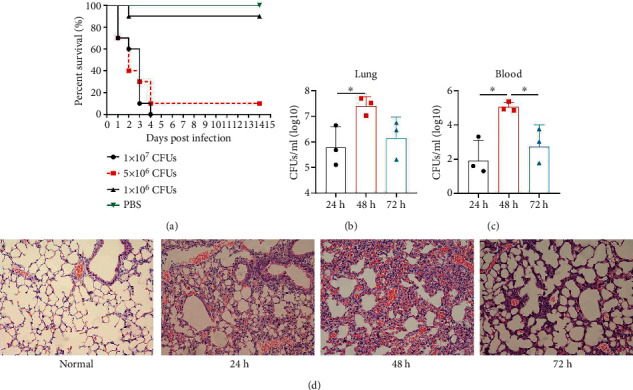
*K. pneumoniae* strain YBQ induced lethal pneumonia. (a) Group-randomized BALB/c mice (*n* = 10) were intratracheally inoculated with 1 × 10^7^, 5 × 10^6^^,^ and 1 × 10^6^ CFU of *K. pneumoniae* YBQ, and survival was monitored for 14 days. Bacterial burden (*n* = 3) of lungs (b) and blood (c) were detected 24, 48, and 72 hours post infection. (d) Histopathology analysis (200×) of lungs was captured 24, 48, and 72 hours post infection. Bacterial burdens were presented as means ± SEM.

The virulence of *K. pneumoniae* clinical strains isolated from the First Hospital Affiliated to Army Medical University (Southwest Hospital) was determined previously [23]. To confirm the virulence of *K. pneumoniae* YBQ in mice from different batch, BALB/c mice were challenged with different dosages of *K. pneumoniae* YBQ. The survival in each group was continuously monitored in 14 days post challenge. 1 × 10^6^ CFUs of YBQ did not exhibit significant lethal effect compared with PBS, but induced observable pneumonia-related symptoms. All mice challenged with 1x10^7^ CFUs of YBQ died in 4 days, one mouse survived from pneumonia induced by 5 × 10^6^ CFUs (Figure 1(a)), these data were in consistent with our previous findings [23].

After confirming the virulence of different dosages of YBQ, 1 × 10^6^ CFUs YBQ was chosen to construct sublethal *K. pneumoniae* pneumonia model. To better understand the host response to *K. pneumoniae* infection, bacterial burden and histopathological characteristics at different time points post infection were assessed. Bacterial burden were peaked at 48 hours post infection and then decreased over time (Figures 1(b) and 1(c)), which suggests that the host immune system was able to control the infection when infected with low dose of *K. pneumoniae*. Consistent with bacterial burden, pulmonary inflammation and injury also reached a most severe degree at 48 hours post infection (Figure 1(d)). Taken together, 48 hours post infection is a critical time point of host immune system to control the *K. pneumoniae* pulmonary infection.

### 3.2. Pulmonary Gene Expression Profile in Response to Sublethal *K. Pneumoniae* Pneumonia

**Figure 2:**
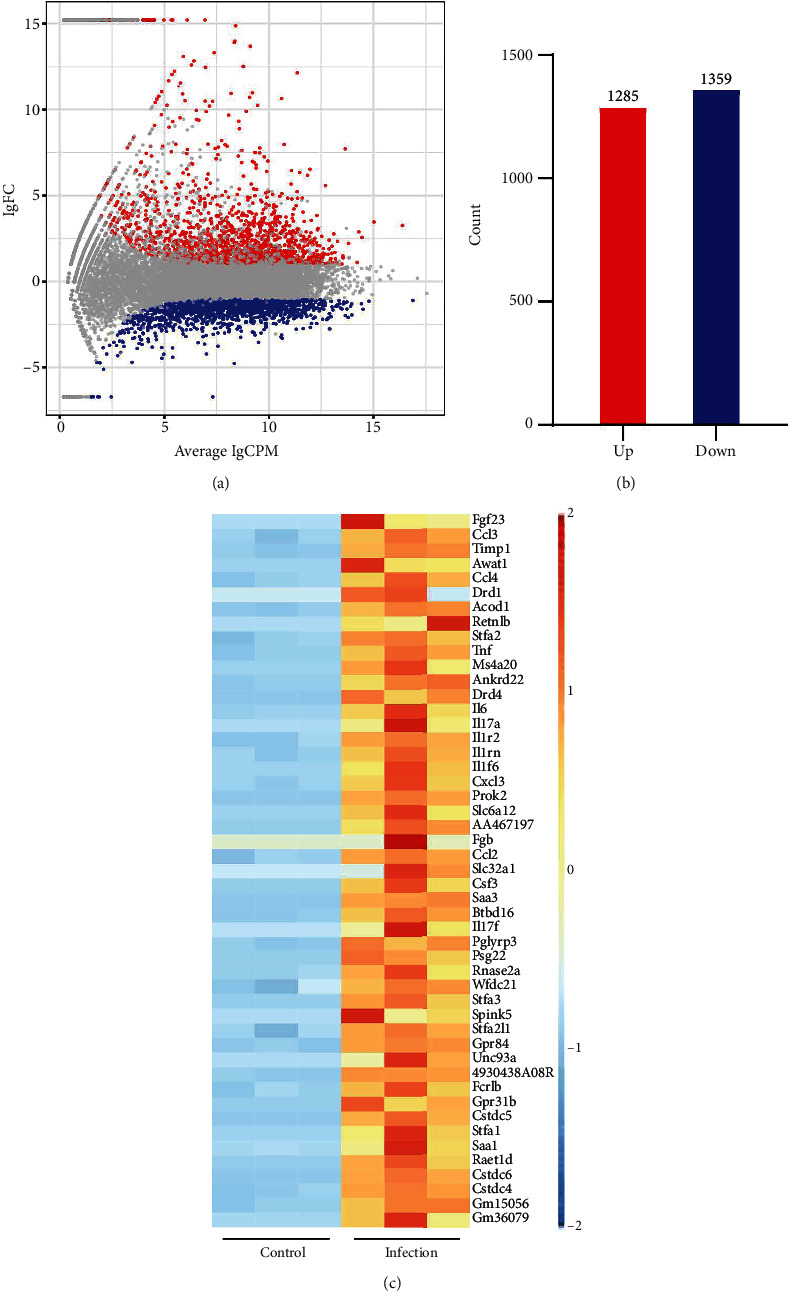
Differentially expressed genes between *K. pneumoniae*-infected lungs (48 hours post infection) and normal lungs. (a) Volcano plot of differentially expressed genes. Differentially expressed genes were shown in red plots (>2 fold upregulated), blue plots (>2 fold downregulated), and gray plots (nonsignificant difference). (b) Counts of upregulated (red) and downregulated (blue) genes. (c) Heatmap of top 50 upregulated genes sorted by fold change.

After establishing the sublethal pneumonia model of *K. pneumoniae* YBQ, lungs from mice challenged with 1 × 10^6^ CFUs of YBQ were collected at 48 hours post infection. Total RNA extracted from these lungs was performed with transcriptome analysis. In RNA-seq analysis, the number of total reads of control group was 43543288, 40817300, and 42651910, and that of infection group was 46551494, 46144342 and 46546712. Q20 of control group was 97.58%, 97.68% and 97.49% and which was 97.7%, 97.71%, and 97.08% for infection group. Q30 of control group was 93.93%, 94.17% and 93.54%, and was 94.22%, 94.32% and 92.64% for infection group. Volcano plot showed the distribution and significant differences in gene transcription (Figure 2(a)). At 48 hours post infection, 1285 genes were unregulated whereas 1359 genes were downregulated (Figure 2(b)), the details of differentiated genes were listed in Tables S1 and S2. The top 50 upregulated genes were presented as heatmap, and several chemokines were enriched in this gene list, including *ccl3*, *ccl4*, *cxcl3*, and *ccl2*. Besides, immune response associated cytokines, *il-17a*, *il-17f*, *il-6*, and *tnf*, were also upregulated to a highly significant extent (Figure 2(c)). Interestingly, the top 50 upregulated genes embodied several cysteine protease inhibitors (*stfa1*, *stfa2*, and *stfa3*) and potential cysteine-type endopeptidase inhibitors (*cstdc4, cstdc5*, and *cstdc6*), which play important roles in antigen processing and presentation [25].

### 3.3. GO Analysis of Significant Differentiated Genes of *K. Pneumoniae*-Infected Lungs

To better understand the biological relevance of significant differentiated genes, Gene Ontology (GO) enrichment analysis of up- and downregulated genes was performed, respectively. The enrichment was grouped into 3 classes, including biological process, cellular component, and molecular function.

**Figure 3:**
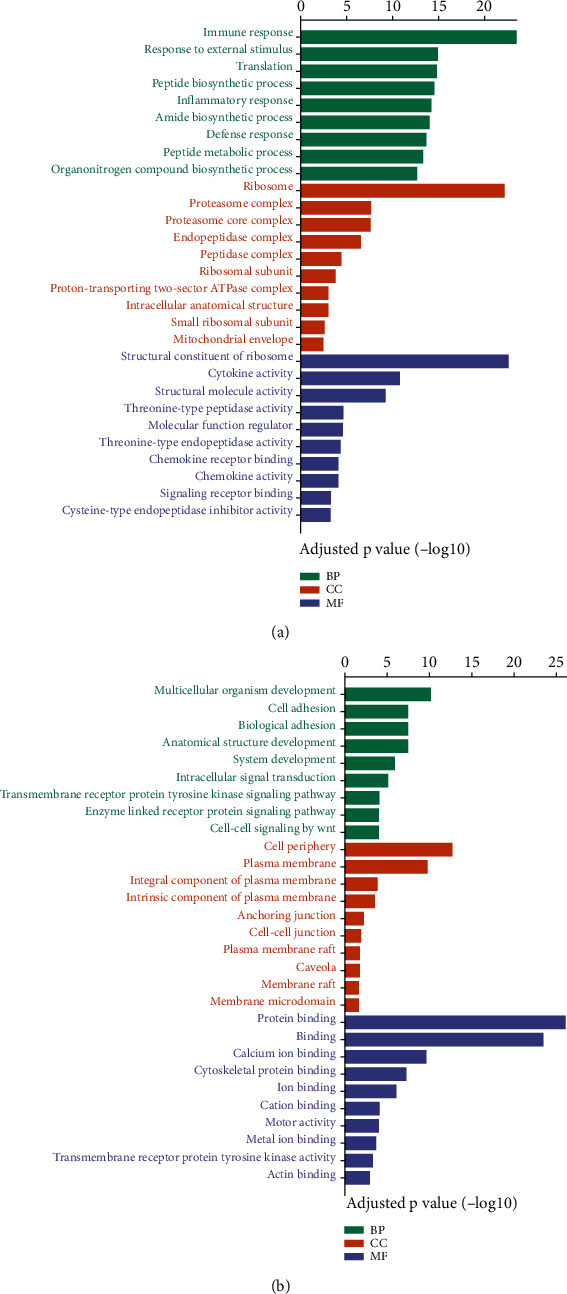
Gene Ontology (GO) enrichment analysis of differentially expressed genes during *K. pneumoniae* infection. Biological process (BP), cellular component (CC), and molecular function (MF) of upregulated (a) and downregulated (b) genes between *K. pneumoniae*-infected lungs vs. normal lungs. The length of the bar represents -log10 adjusted *P* value.

The predominant upregulated genes enriched in the biological process were involved in immune response, response to external stimulus, inflammatory response, defense response, and other synthetic processes. Upregulated genes were mainly parts of the ribosome, proteasome, endopeptidase, peptidase, which was consistent with their role in cytokines activity, chemokine activity, chemokine receptor binding, and signaling transduction (Figure 3(a)).

The downregulated genes mainly participant in biological process indicated the inhibition of cell adhesion, biological adhesion, multicellular organism development, anatomical structure development, and system development. As expected in cellular component part, significant downregulation occurred in the junctions among the cells and plasma membrane components, which may provide the condition for immune cell infiltration and bacterial dissemination. Interestingly, several types of bindings were inhibited by the infection of *K. pneumoniae* (Figure 3(b)). Taken together, transcriptome systematic alteration induced by *K. pneumoniae* infection indicated the coordination between non-immune response and immune response.

### 3.4. KEGG Analysis of Significant Differentiated Genes of *K. Pneumoniae*-Infected Lungs

**Figure 4:**
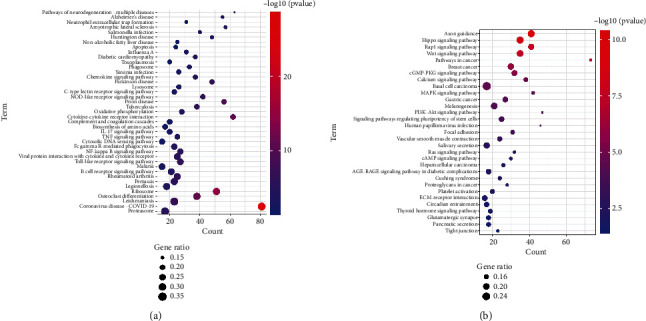
KEGG pathway classification of differentially expressed genes induced by *K. pneumoniae* infection. KEGG terms of upregulated (a) and downregulated (b) genes in *K. pneumoniae*-infected lungs at 48 hours post infection.

To detail the signaling pathways involved in *K. pneumoniae* pulmonary infection, Kyoto Encyclopedia of Genes and Genomes (KEGG) pathway classification of up- and downregulated genes was carried out, respectively. Upregulated genes were enriched at several immune associated pathways, including B cell receptor signaling pathway, Toll-like receptor signaling pathways, NF-*κ*B signaling pathways, Fc*γ*R mediated phagocytosis, cytosolic DNA sensing pathways, TNF pathways, IL-17 pathways, complement and coagulation cascades, NOD-like receptor signaling pathways, C-type lectin receptor signaling pathways, phagosome, lysosome, and neutrophil extracellular trap formation (Figure 4(a)). These signaling pathways depicted the recognition, engulfment, and digestion of *K. pneumoniae* by host immune system. Downregulated genes were enriched in some carcinoma pathways, suggesting inhibition of proliferation, promotion of differentiation, and consistence with activation of immune cells. Several types of cell junctions were decreased, which may promote immune cell infiltration (Figure 4(b)). As depicted in GO analysis, these data elucidated the synergy of immune system and non-immune system.

### 3.5. Activation of Pulmonary IL-17 and TNF Signaling Pathways Induced by *K. Pneumoniae* Infection

IL-17 pathway has been identified as a critical defense factor against bacterial infection [26–28]. According to RNA-seq analysis, transcription of IL-17A, IL-17F, and IL-17RA were significantly elevated during *K. pneumoniae* infection (Figure S1A). Besides, KEGG analysis of transcriptome data were enriched into IL-17 pathways (*P* < 0.00004871) and several critical IL-17 pathway downstream genes were upregulated, including chemokines (*cxcl1*, *cxcl2, cxcl5*, *cxcl10*, *ccl2*, *ccl7*, and *ccl20*), cytokines (*il-6*,*TNF-α*, and *G-CSF*), and antimicrobial factors (*MUC5AC*, *S100A8*, *S100A9*, and *LCN2*). These data suggest that IL-17 pathway may play an essential role in eradication of *K. pneumoniae*.

TNF pathway was also highlighted in KEGG analysis (*P* < 9.212 × 10^−7^) and several critical TNF pathway downstream genes were upregulated, including *csf1*, *fas*, and *nod2*. (Figure S1B). TNF-*α* is required in immune defense against Mycobacterium tuberculosis in mice [29] via inducing bactericidal granulomas [30]. Besides, anti-TNF-*α* therapy rendered patients susceptible to bacterial infection [31]. Given this, it is not surprising that TNF pathway was activated during *K. pneumoniae* infection and may play an essential role in clearing the infection.

### 3.6. Immune Cell Typing of Infected Lung

**Figure 5:**
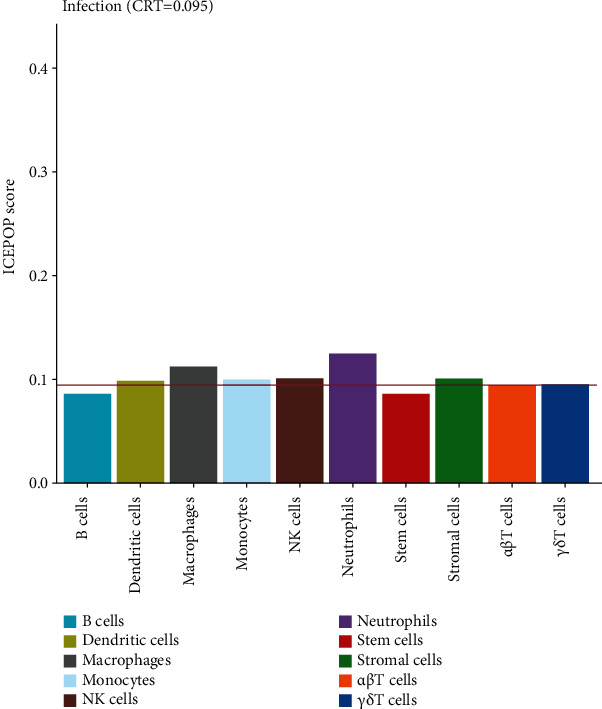
Immune cell population of differential gene expression. The height of the histogram (ICEPOP Score) represents the extent of cell response. Cell type response threshold (CR, red line) is a threshold to determine whether a cell type is responding or not.

ICEPOP (Immune CEll POPulation) is the method for estimating immune cell population in the expressed genes and enabling analysis of differentially expressed genes [32]. To further elucidate the major immune cell types responsible for reversing the exacerbation of *K. pneumoniae* infection, ICEPOP was applied to analysis the differentially expressed genes. The cell type, with ICEPOP score over the cell type response threshold (CRT), was considered as responsive to the infection. As shown in Figure 5, neutrophils and macrophages had the highest and second highest ICEPOP scores, respectively, while monocytes, dendritic cells, stromal cells, NK cells, and *γδ*T cells had relatively lower response. These data suggest that neutrophils may be the dominant cell subtype required for eradicating *K. pneumoniae* infection, which is consistent with our previous report [23].

### 3.7. Validation of Key Differentially Expressed Genes

**Figure 6:**
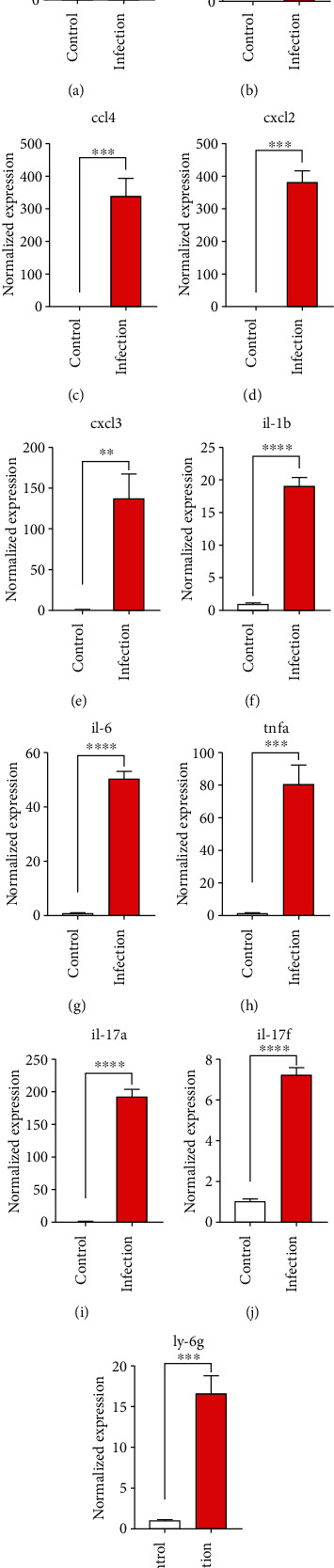
Validation of mRNA expression level of selected significant chemokines, cytokines, and cell markers. BALB/c mice were intratracheally inoculated with 1x10^6^ CFUs *K. pneumoniae* YBQ for 48 hours or not. Chemokines *ccl2* (a), *ccl3* (b), *ccl4* (c), *cxcl2* (d), *cxcl3* (e); cytokines *il-1b* (f), *il-6* (g), *tnfa* (h), *il-17a* (i), *il-17f* (j); neutrophil marker *ly-6g* (k). Unpaired *t*-test was performed to calculate the significance of difference between infected lungs and normal lungs (*n* = 3). ^∗∗^, *P* < 0.01; ^∗∗∗^, *P* < 0.001; ^∗∗∗∗^, *P* < 0.0001.

To confirm the significance of differentially expressed genes, 11 genes were evaluated by quantitative real-time PCR, including *ccl2*, *ccl3, ccl4*, *cxcl2*, *cxcl3*, *il-1b*, *il-6*, *tnf-a*, *il-17a, il-17f*, and *ly-6*g (Figure 6). Transcription of chemokines responsible for recruiting monocytes or/and neutrophils (*ccl2*, *ccl3, ccl4*, *cxcl2*, and *cxcl3*) were boosted to an extent ranging from 58.22 to 425.9-folds. Three upregulated cytokines, *il-1b* (18.09 ± 0.7539-fold change), *il-6* (49.40 ± 1.641-fold change), and *tnf-a* (79.59 ± 6.921-fold change), indicated significant pulmonary inflammation levels post infection. The activation of IL-17 signaling pathways was also validated by *il-17a* (191.4 ± 6.152 -fold change) and *il-17f* (6.224 ± 0.2050-fold change), suggesting that IL-17A played a dominant role in activation of IL-17 pathways. Neutrophils recruitment was confirmed indirectly by *ly-6g* (15.58 ± 1.240-fold change), a surface marker of neutrophils. Taken together, key results of transcriptome analysis were confirmed by quantitative real-time PCR.

## 4. Discussion

In this study, a sublethal pneumonia caused by *K. pneumoniae* was carried out and bacteria burden was peaked at 48 hours post infection, another study showed that body weight loss of mouse infected with sublethal dose of four clinical strains was also peaked at 48 hours post infection [20]. These data suggested that murine immune systems failed to control the proliferation of *K. pneumoniae* clinical strains isolated from patients in 48 hours post infection. Here, our data revealed the global immune response to *K. pneumoniae* clinical strains infection at turning point via transcriptome analysis.

*Klebsiella pneumoniae* strain YBQ was newly isolated from the sputum of a patient with severe pneumonia, and its genome was sequenced [24]. CPS genotyping by PCR detection of serotype-specific alleles at wzy and wzx loci [33] showed that YBQ belongs to neither K1 nor K2 serotypes. However, it exerted high lethal effects on a mouse model, and the virulence was comparable or even higher than some K1 strains, such as YYD [23]. Given the dominant prevalence of K1 and K2 type strains, a nonK1 or -K2 type strain harbors high pathogenicity indicate that some other types of capsular can also greatly contribute to the pathogenicity of *K. pneumoniae*.

In this study, several cysteine cathepsin inhibitors (*stfa1*, *stfa2*, and *stfa3*) were significantly upregulated upon *K. pneumoniae* infection. Cysteine cathepsin was initially identified as proteases responsible for the bulk proteolysis of intracellular and extracellular proteins in the acidic environment of the endosomal/lysosomal compartment [34]. On the other side, phagolysosome functions as a dominant bacterial eradication site of phagocytes [35]. Cysteine cathepsin inhibitors regulated the immune response by upregulating anti-inflammatory cytokines and downregulating proinflammatory cytokines to modulate T-cell responses and promote macrophage polarization [36]. Our data suggests inhibition rather than activation of cysteine cathepsin may be required for effective immune response to *K. pneumoniae* pulmonary infection. *Cstdc4, cstdc5*, and *cstdc6* were termed as cysteine-type endopeptidase inhibitors by GO and also listed into top 50 upregulated genes. However, their function in anti*K. pneumoniae* has not been studied previously. These immune factors may play an essential role in effective eradication of *K. pneumoniae.*

Extensive studies have defined the importance of neutrophils in clearance of *K. pneumoniae* infection [37, 38]. Neutrophils recruitment partially depends on activation of signaling pathways network including MyD88, TRIF, interleukin-1 receptor (IL-1R), Toll-like receptor 4 (TLR4), and leukotriene B4 and positively correlated with defense against *K. pneumoniae* [16, 39–41]. However, deficiency of C-type lectin receptors increased neutrophil recruitment but impaired host eradication of *K. pneumoniae* [42]. Fully antibacterial function of neutrophils required specific microenvironment, including aid of other cells, cytokines, and nutrition molecules. In this study, neutrophil response was identified by ICEPOP analysis and pulmonary *ly-6g* transcription upregulation, which was consistent with our previous results of neutrophil depletion assay [23].

CCR2+ monocytes (Ly6C^hi^ and CD11b), termed inflammatory monocytes, have been reported as a dominant cell type in eradicating particular *K. pneumoniae* strain but dispensable participation in clearance of other strains [20]. On the other side, recruitment of monocytes required CCL2-CCR2 mediated signaling pathways [43]. In this study, the results showed that CCL2 was remarkably elevated and monocyte response was identified by ICEPOP, suggesting CCR2+ monocytes may play a role in resolution of pulmonary infection of YBQ. TH17 cells were reported to confer protection under conditions of transplant immunosuppression [44], and innate lymphocytes 3-(ILC3s-) producing IL-17A enhances eradication of *K. pneumoniae* by CCR2+ monocytes via IL-17R highly expressed on CCR2+ monocytes surface [20]. In this study, differentially expressed genes were significantly enriched into IL-17 signaling pathway, and upregulated transcription level of IL-17A and IL-17F were confirmed with rt-PCR. Apart from enhancement of CCR2+ monocytes antibacterial function, neutrophils recruitment also requires IL-17A [45]. Data of this study further support the critical role of IL-17, CCR2+ monocytes, and neutrophils in host defense against *K. pneumoniae*.

Macrophages were identified as secondary significant responding cells by ICEPOP in this study. Alveolar macrophages engulf *K. pneumoniae* by CD36, a scavenger receptor [46]. But numbers of alveolar macrophages were not altered during *K. pneumoniae* infection. Macrophages take part in immune protection against this pathogen in the intestine with the development of bacteroidetes and IL-36 signaling [47]. However, to our knowledge, there is lack of studies directly investigating the role of macrophages in clearance of *K. pneumoniae.*

There are also some limitations in our study. First, transcriptome analysis only unraveled the transcriptional alteration of effector proteins, but actual quantity and activation of these proteins required further determination. Second, the results of ICEPOP analysis also needed further elucidation and detailed investigation with flow cytometry.

## 5. Conclusion

In this study, we detected the transcriptional alteration of genes from the lungs of mice 48 hours post infection with a newly isolated *K. pneumoniae* clinical strain YBQ and analyzed with KEGG, GO, and ICEPOP. We found that upregulated transcription level of numerous cytokines and chemokines was coordinated with IL-17 and TNF signaling pathways, and neutrophils may be the dominant cell subtype required for eradicating *K. pneumoniae* infection. In addition, we validated the critical gene alterations by rt-PCR. Further analysis of these critical genes and immune cells are required to clarify their mechanism during the infection, thereby providing new insights into the treatment of *K. pneumoniae* infection.

## Data Availability

Raw data files of RNA-seq have been deposited in the NCBI Gene Expression Omnibus under accession number GEO: GSE171048.

## Supplementary Materials

Supplementary Materials Figure S1 Map of KEGG enrichment of IL-17 (A), TNF (B) signaling pathways in response to *K. pneumoniae* pneumonia. Red color, green color, and white color represents upregulated genes, downregulated genes, and unchanged genes, respectively. Table S1 Upregulated gene list of control vs. infection. Inf indicates zero quantitative values in the control group. Table S2 Downregulated gene list of control vs. infection. Inf indicates zero quantitative values in the control group.
