# Supplementary figures and images for: Host Immune Response to Clinical Hypervirulent Klebsiella pneumoniae Pulmonary Infections via Transcriptome Analysis
Source: J Immunol Res. 2022 Sep 20;2022:5336931. doi: 10.1155/2022/5336931 (PMC9553456; doi:10.1155/2022/5336931)

## IL-17 SIGNALING PATHWAY

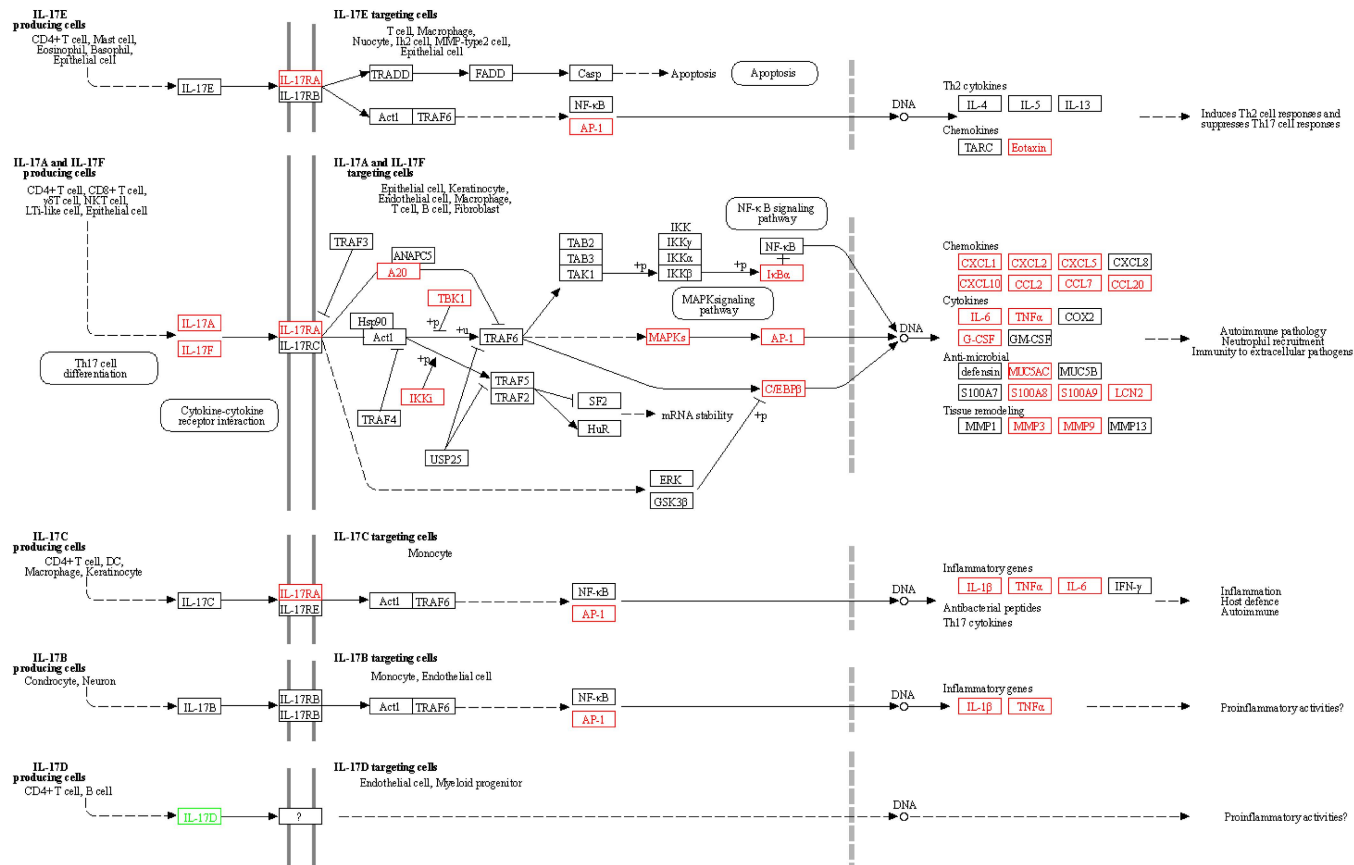

# B

## TNF SIGNALING PATHWAY

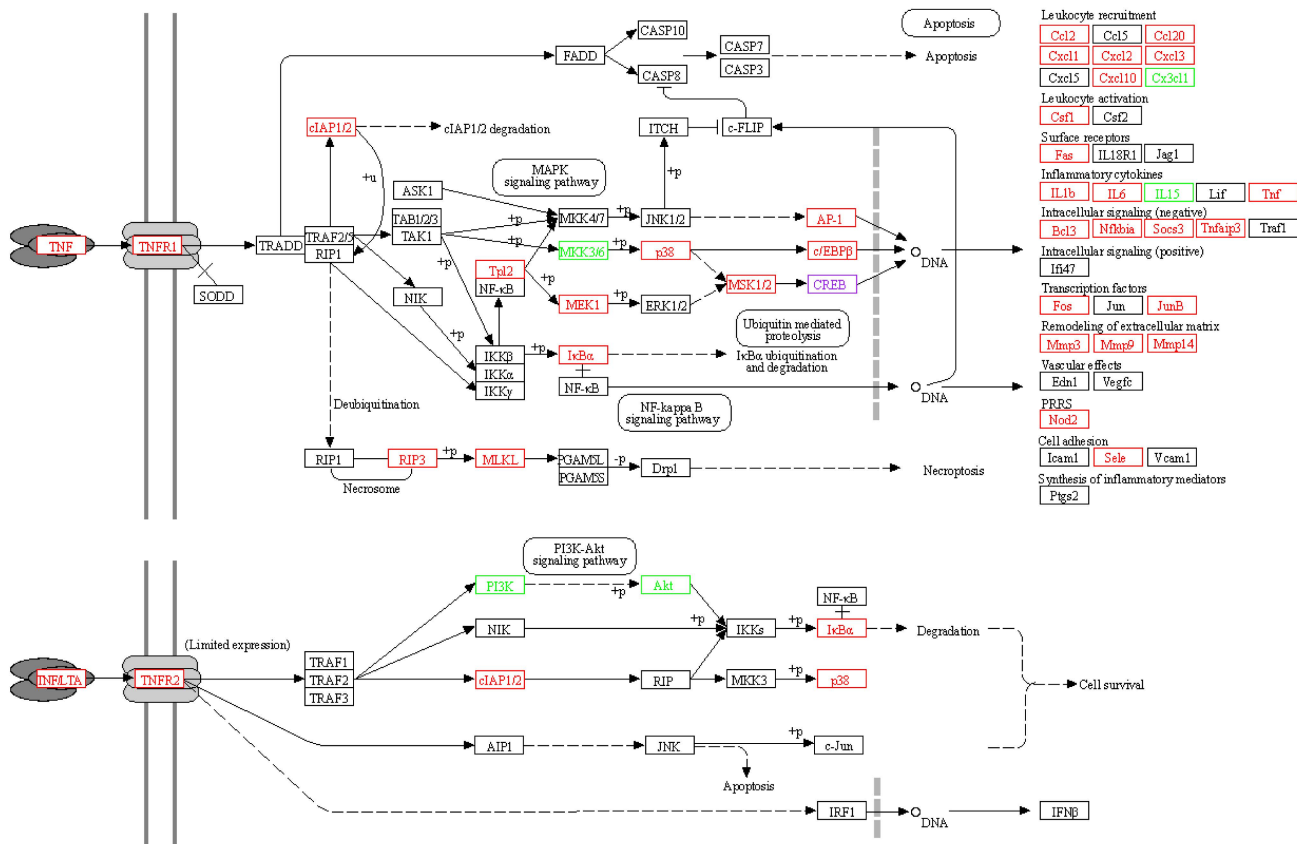

Supplement: Supplementary Materials — Figure S1 Map of KEGG enrichment of IL-17 (A), TNF (B) signaling pathways in response to K. pneumoniae pneumonia. Red color, green color, and white color represents upregulated genes, downregulated genes, and unchanged genes, respectively. Table S1 Upregulated gene list of control vs. infection. Inf indicates zero quantitative values in the control group. Table S2 Downregulated gene list of control vs. infection. Inf indicates zero quantitative values in the control group. [file 5336931.f1.zip › FigureS1.pdf]
